# Cellular Metabolomics Reveal the Mechanism Underlying the Anti-Atherosclerotic Effects of Aspirin Eugenol Ester on Vascular Endothelial Dysfunction

**DOI:** 10.3390/ijms20133165

**Published:** 2019-06-28

**Authors:** Mei-Zhou Huang, Xiao-Rong Lu, Ya-Jun Yang, Xi-Wang Liu, Zhe Qin, Jian-Yong Li

**Affiliations:** Key Lab of New Animal Drug Project of Gansu Province, Key Lab of Veterinary Pharmaceutical Development of Ministry of Agriculture, Lanzhou Institute of Husbandry and Pharmaceutical Sciences of CAAS, Lanzhou 730050, China

**Keywords:** aspirin eugenol ester, vascular endothelium, cell metabolomics

## Abstract

Aspirin eugenol ester (AEE) possesses anti-thrombotic, anti-atherosclerotic and anti-oxidative effects. The study aims to clarify the mechanism underlying the anti-atherosclerotic effects of AEE on vascular endothelial dysfunction. Both the high-fat diet (HFD)-induced atherosclerotic rat model and the H_2_O_2_-induced human umbilical vein endothelial cells (HUVECs) model were used to investigate the effects of AEE on vascular endothelial dysfunction. UPLC/QTOF-MS coupled with a multivariate data analysis method were used to profile the variations in the metabolites of HUVECs in response to different treatments. Pretreatment of HUVECs with AEE significantly ameliorated H_2_O_2_-induced apoptosis, the overexpression of E-selectin and VCAM-1, and the adhesion of THP-1 cells. Putative endogenous biomarkers associated with the inhibition of endothelial dysfunction were identified in HUVECs pretreated with AEE in the absence or presence of H_2_O_2_, and these biomarkers were involved in important metabolic pathways, including amino acid metabolism, carbohydrate metabolism, and glutathione metabolism. Moreover, in vivo, AEE also significantly reduced vascular endothelial dysfunction and decreased the overexpression of VCAM-1 and E-selectin. Based on our findings, the mechanism underlying the anti-atherosclerotic effects of AEE might be related to a reduction in vascular endothelial dysfunction mediated by ameliorating alterations in metabolism, inhibiting oxidative stress, and decreasing the expression of adhesion molecules.

## 1. Introduction

The endothelium has been regarded as a pivotal regulator of vascular homeostasis [1,2]. Dysfunctional, activated endothelium induced by various stimuli may increase leukocyte adhesion and the production of cytokines and growth factors, which would promote the development of atherosclerosis, a thrombus and other cardiovascular diseases [3,4,5,6,7,8]. An appreciation of the key role of the endothelium in the cardiovascular disease process has led to the development of new drugs to regulate its function [9,10].

AEE is synthesized by combining aspirin with eugenol based on the prodrug principal [11]. AEE possesses various pharmacological activities, including anti-thrombus, anti-atherosclerotic, and anti-oxidative properties [12,13,14,15,16]. The mechanism by which AEE regulates cardiovascular diseases remains unknown, although many in-vivo studies of AEE have been well documented by our group [15,17,18]. In-vitro investigations of AEE coupled with the application of new technical methods will be beneficial for clarifying the mechanism of action of AEE.

Metabolism, a ubiquitous biological process, reflects the state of the cells, tissue, organs and whole body in real time. Since metabolomics facilitates the high-throughput, global, and comprehensive characterization of small molecule metabolites in a biological system and their changes, it has been adopted to diagnose diseases, develop drugs and evaluate pharmacodynamics [19,20,21,22,23]. Recently, cellular metabolomics has been used to systematically investigate the small-molecule metabolites in specific cells [24,25]. Cellular metabolomics is also an ideal technical method to probe the effects of AEE on vascular endothelial cell dysfunction. In the present study, the effects of AEE on vascular endothelial cell dysfunction were investigated in vitro, and differences in the cellular metabolite levels between untreated human umbilical vein endothelial cells (HUVECs) and cells with H_2_O_2_ and/or AEE were evaluated to further understand the mechanism underlying the anti-atherosclerotic effects of AEE on vascular endothelial dysfunction. Moreover, the anti-atherosclerotic mechanism of AEE was confirmed in a rat model of HFD-induced atherosclerosis.

## 2. Results

### 2.1. AEE Reduced the H_2_O_2_-Induced Apoptosis of HUVECs

**Figure 1:**
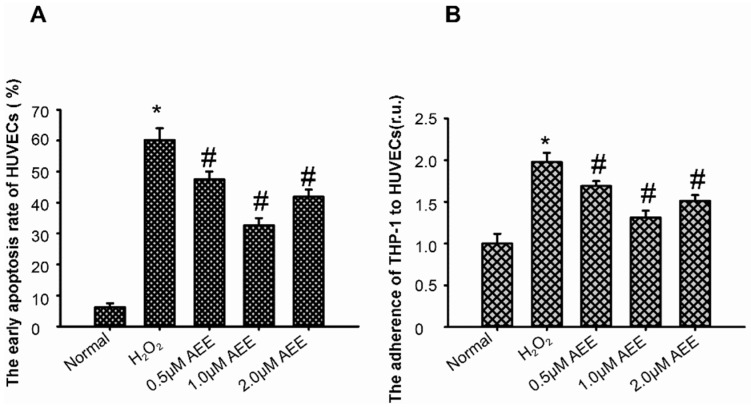
AEE reduced the apoptosis of HUVECs and decreased the H_2_O_2_-induced adherence of THP1 cells to HUVECs. (**A**) AEE reduced the apoptosis of HUVECs induced by H_2_O_2_. Values are presented as the means ± SD where applicable (*n* = 6). * *p* < 0.05 compared with the normal group; ^#^ *p* < 0.05 compared with the H_2_O_2_ group. (**B**) AEE decreased the H_2_O_2_-induced adherence of THP1 cells to HUVECs. Values are presented as the means ± SD where applicable (*n* = 6). All data were normalized to the corresponding control and reported in relative units (r.u.).

In order to detect the effects of AEE on H_2_O_2_-induced HUVECs, the H_2_O_2_-induced oxidative injury model of HUVECs was established. After treating HUVECs with 200 µM H_2_O_2_ for 22 h, the percentage of early apoptotic cells was approximately 60%. Pre-incubation of HUVECs with different concentrations of AEE (0.5, 1, or 2 µM) for 24 h markedly decreased the percentage of early apoptotic HUVECs. Moreover, the pretreatment with 1 µM AEE exerted the best effect (Figure 1A). The results suggested that AEE could effectively prevent H_2_O_2_-induced oxidative injury of HUVECs.

### 2.2. AEE Ameliorated Alterations in the Biochemical Profile

To confirm whether the effects of AEE on vascular endothelial in vivo and in vitro are similar, the HFD-induced rat model of atherosclerosis was used to investigate anti-vascular endothelial dysfunction effects of AEE. The physiological biochemical parameters and AGE level of HFD-fed rats were significantly disturbed, as shown in Table 1. The levels of LDL, TCH, and AGEs were markedly increased (*p* < 0.05), and the level of HDL was significantly decreased (*p* < 0.05) compared with the normal group. Meanwhile, the changes in the biochemical profile and AGE levels were significantly ameliorated in rats pretreated with AEE (54 mg/kg body weight).

### 2.3. AEE Suppressed the Oxidative Injury-Induced Imbalance in Vascular Adhesion

**Figure 2:**
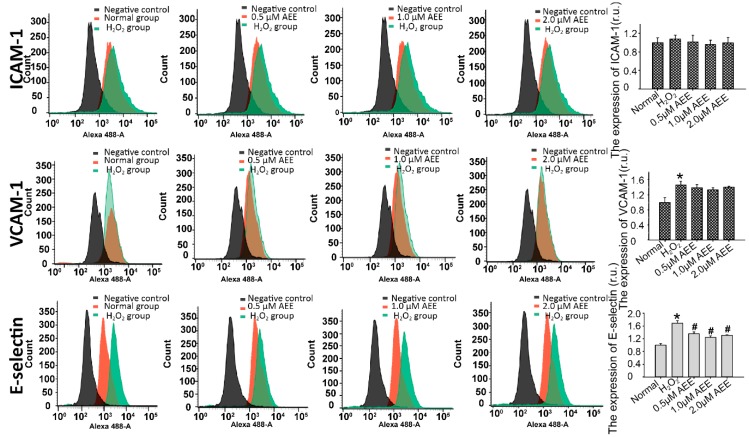
AEE decreased H_2_O_2_-induced E-selection expression in HUVECs. Values are presented as the means ± SD where applicable (*n* = 8). * *p* < 0.05 compared with the normal group; ^#^ *p* < 0.05 compared with the H_2_O_2_ group. All data were normalized to the corresponding control and in relative units (r.u.).

The imbalance in vascular adhesion is a vital characteristic of vascular endothelial dysfunction. After treating HUVECs with 200 µM H_2_O_2_ for 22 h, the adherence of THP-1 cells to HUVECs was significantly increased in vitro. The pre-incubation with AEE (0.5, 1.0, or 2.0 µM) significantly attenuated the adherence of THP-1 cells to HUVECs (Figure 1B). The expression of VCAM-1, ICAM-1, and E-selectin on HUVECs among the different treatment groups was detected using flow cytometry. As shown in Figure 2, the treatment of HUVECs with 200 µM H_2_O_2_ for 22 h significantly increased VCAM-1 and E-selectin expression. However, in the cells pretreated with AEE (0.5, 1.0, or 2.0 µM), the stimulatory effect of H_2_O_2_ on malondialdehyde (MDA) levels was significantly reduced. ICAM-1 expression was not different among the different treatment groups, and VCAM-1 expression was also not different between the AEE pretreatment group and H_2_O_2_-induced group. These findings suggested that AEE could effectively suppress the oxidative injury-induced imbalance in vascular adhesion.

**Figure 3:**
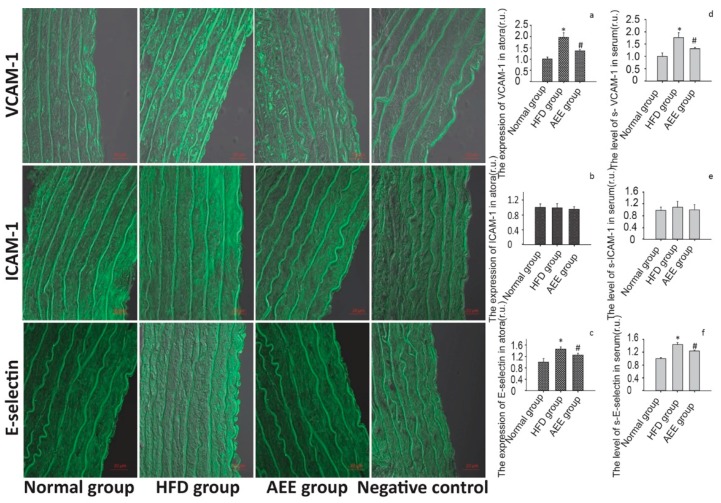
AEE reduced the expression of adhesion molecules in rats fed the HFD. (**a**–**c**): AEE reduced VCAM-1 and E-selectin expression in the aorta. Values are presented as the means ± SD where applicable (*n* = 8). * *p* < 0.05 compared with the normal group; ^#^ *p* < 0.05 compared with the HFD group. All data were normalized to the corresponding control and reported in relative units (r.u.). (**d**–**f**): AEE decreased the serum sVCAM-1 and sE-selectin levels. Values are presented as the means ± SD where applicable (*n* = 8). * *p* < 0.05 compared with the normal group; ^#^ *p* < 0.05 compared with the HFD group. All data were normalized to the corresponding control and reported in relative units (r.u.).

As shown in Figure 3, rats fed the HFD for 12 weeks exhibited a significant increase in VCAM-1 and E-selectin expression in the aorta in vivo compared with the normal group, while ICAM-1 expression in the aorta was not different between the HFD group and normal group. In rats pretreated with AEE (54 mg/kg body weight), the HFD-induced overexpression of VCAM-1 and E-selectin was significantly reduced. Moreover, the serum levels of sVCAM-1 and sE-selectin were significantly increased in HFD-fed rats. In the presence of AEE, the levels of sVCAM-1 and sE-selectin were significantly reduced compared with the HFD-fed groups. ICAM-1 levels were not significantly different among the different treatment groups. The results in vivo also proposed that AEE could effectively attenuate imbalance in vascular adhesion.

### 2.4. Metabolomics Analysis of Cells and Cell Culture Supernatants

#### 2.4.1. Metabolomics Analysis of Cell Culture Supernatants

**Figure 4:**
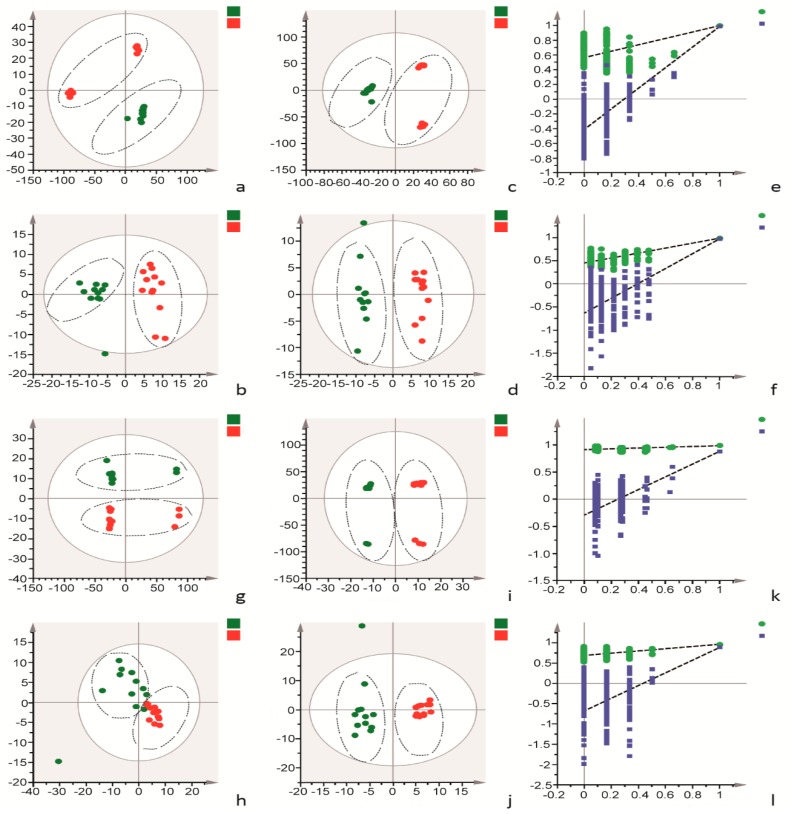
Multivariate analyses data from the UPLC-Q-TOF/MS analysis of cells and cell culture supernatants in the absence of H_2_O_2_. (**a**,**b**): PCA score plots based on cell culture supernatant metabolic profiles of the normal and AEE groups in positive and negative modes, ESI+: R^2^ = 0.773, ESI−: R^2^ = 0.602. (**c**,**d**): OPLS-DA score plots of normal and AEE groups in positive and negative modes, ESI+: R^2^X = 0.773, R^2^Y = 0.993, Q^2^ = 0.982; ESI−: R^2^X = 0.442, R^2^Y = 0.985, Q^2^ = 0.971. (**e**,**f**): Permutation test of the OPLS-DA model, ESI+: the intercepts of R^2^ = 0.512 and Q^2^ = −0.424, ESI−: the intercepts of R^2^ = 0.469 and Q^2^ = −0.615. (**g**,**h**): PCA score plots based on cellular metabolic profiles of the normal and AEE groups in positive and negative modes, ESI+: R^2^ = 0.778, ESI−: R^2^ = 0.643. (**i**,**j**): OPLS-DA score plots of the normal and AEE groups in positive and negative modes, ESI+: R^2^X = 0.795, R^2^Y = 0.99, Q^2^ = 0.886; ESI−: R^2^X = 0.542, R^2^Y = 0.965, Q^2^ = 0.885. (**k**,**l**): Permutation test of the OPLS-DA model, ESI+: the intercepts of R^2^ = 0.923 and Q^2^ = −0.225, ESI−: the intercepts of R^2^ = 0.696 and Q^2^ = −0.72. Z: normal group; B: AEE group.

**Figure 5:**
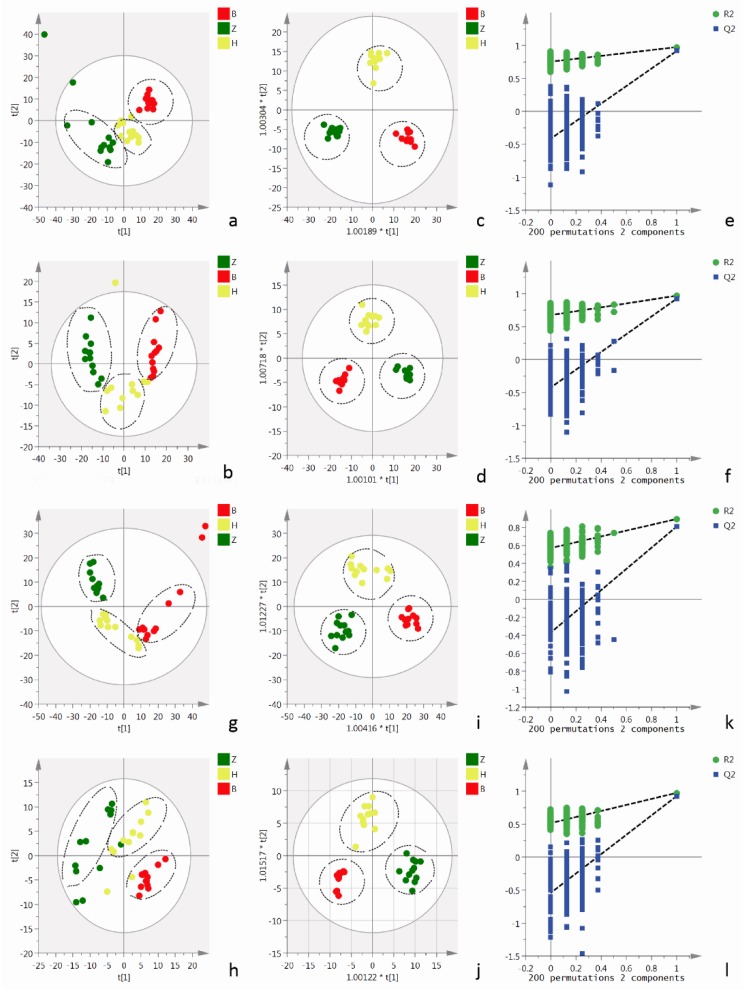
Multivariate analyses of data from the UPLC-Q-TOF/MS analysis of cell and cell culture supernatants stimulated with H_2_O_2_. (**a**,**b**): PCA score plots based on cell culture supernatant metabolic profiles of the normal, H_2_O_2_ and AEE groups in positive and negative modes, ESI+: R^2^ = 0.527, ESI−: R^2^ = 0.593. (**c**,**d**): OPLS-DA score plots of the normal, H_2_O_2_ and AEE groups in positive and negative modes, ESI+: R^2^X = 0.392, R^2^Y = 0.975, Q^2^ = 0.862; ESI−: R^2^X = 0.511, R^2^Y = 0.966, Q^2^ = 0.848. (**e**,**f**): Permutation test of the OPLS-DA model, ESI+: the intercepts of R^2^ = 0.751 and Q^2^ = −0.427, ESI−: the intercepts of R^2^ = 0.675 and Q^2^ = −0.422. (**g**,**h**): PCA score plots based on the cellular metabolic profiles of the normal, H_2_O_2_ and AEE groups in positive and negative modes, ESI+: R^2^ = 0.56, ESI−: R^2^ = 0.668. (**i**,**j**): OPLS-DA score plots of normal and AEE groups in positive and negative modes, ESI+: R^2^X = 0.4, R^2^Y = 0.911, Q^2^ = 0.836; ESI−: R^2^X = 0.531, R^2^Y = 0.924, Q^2^ = 0.814. (**k**,**l**): Permutation test of the OPLS-DA model, ESI+: the intercepts of R^2^ = 0.568 and Q^2^ = −0.0.375, ESI−: the intercepts of R^2^ = 0.518 and Q^2^ = −0.545. Z: normal group; B: AEE group; H: H_2_O_2_ group.

The disorder of cell metabolism is an important incentive of imbalance in vascular adhesion. Therefore, the metabolomics of cells and cell culture supernatants was detected among different treatment groups using UPLC-Q-TOF/MS to determine the effect of AEE on the cellular metabolite levels. Representative total ion chromatograms (TICs) of the cells and cell culture supernatants showed good separation and strong sensitivity of the established method (Supplementary materials). Moreover, the number of metabolites is presented in the Supplementary materials. A principal component analysis (PCA) was performed to visualize grouping trends and outliers in the MS data of cell culture supernatants collected in positive and negative mode, respectively. As indicated by the score plots of the first two principal components (t1/t2) shown in Figure 4a,b, the metabolic profiles of cell culture supernatants from the normal group and AEE group in the absence of H_2_O_2_ were clearly separated in positive and negative modes. The metabolic profiles of cell culture supernatants from the normal group, H_2_O_2_ group and AEE group were clearly separated in positive and negative modes (Figure 5a,b). The values for the model parameter R2X, which represent the explanatory ability of the model, were 0.627 and 0.625 in positive and negative modes, respectively, indicating that the data were highly elucidated by the two PCA models.

Orthogonal projections to latent structures discriminate analysis (OPLS-DA) model was constructed and tested with the permutation test to further explore the differences in metabolites among the different treatment groups and the effect of AEE on oxidative injury. In the OPLS-DA analysis, the AEE group and normal group were clearly separated in the absence of H_2_O_2_ (Figure 4c,d), consistent with the previous results of the PCA. Moreover, the AEE group, H_2_O_2_ group and normal group were clearly divided into three regions (Figure 5c,d). As indicated by the validation plots shown in Figure 4e,f and Figure 5e,f, in all OPLS-DA models, permutation tests generated Q2 regression lines with a negative intercept, and all permuted R2 values located to the left of the intercept were lower than the original point to the right of the intercept.

After building the OPLS-DA model, a variable importance analysis was conducted as the key step before the biomarker analysis. The variables in the OPLS-DA were screened with a VIP value greater than 1.0 and *p* < 0.05. Through further identification of putative metabolites, four potential biomarkers were selected and summarized in Table 2.

#### 2.4.2. Cellular Metabolomics Analysis

The results from the cellular metabolomics analysis were similar to the results obtained from cell culture supernatant samples. Typical TICs of cellular extracts in positive and negative modes are shown in Supplementary materials. As shown in the PCA score plots, clear boundaries between the normal group and AEE group were observed in the absence of H_2_O_2_ in both positive and negative modes (Figure 4g,h). A clear separation among the normal, H_2_O_2_ and AEE groups was observed (Figure 5g,h). As shown in score plots of OPLS-DA models (Figure 4i,j and Figure 5i,j), clear compartmentation was also observed among the different treatment groups in the absence or presence of H_2_O_2_. Moreover, none of the OPLS-DA models were overfitted, according to the results of the permutation test, as shown in Figure 4k,l and Figure 5k,l. The Q2 regression line has a negative intercept, and all permuted R2 values located to the left of the intercept were lower than the original point to the right of the intercept.

### 2.5. Identification of the Biomarkers and Pathway Analysis

**Figure 6:**
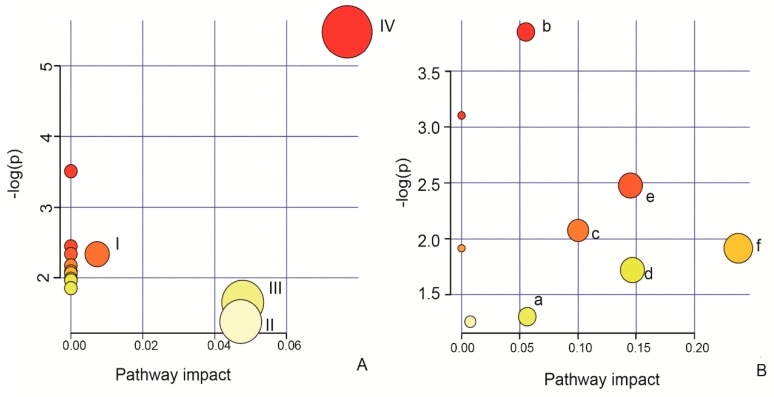
Results of the pathway analysis of the potential biomarkers in cells and cell culture supernatants. (**A**) Effect of AEE on possible metabolic pathways in the absence of H_2_O_2_. I: Phenylalanine, tyrosine and tryptophan biosynthesis; II: tyrosine metabolism; III: cysteine and methionine metabolism; and IV: valine, leucine and isoleucine biosynthesis. (**B**) Effect of AEE on possible metabolic pathways disturbed by H_2_O_2_. a: Aminoacyl-tRNA biosynthesis; b: cysteine and methionine metabolism; c: lysine biosynthesis; d: lysine degradation; e: riboflavin metabolism; and f: glutathione metabolism.

After building the OPLS-DA model, a variable importance (VIP) analysis was conducted as the key step before the biomarker analysis. The potential biomarkers were screened with a VIP value > l and *p* < 0.05. Then, an analysis of metabolomics pathways was performed with MetaboAnalyst 3.0 software to identify and visualize the most relevant metabolic pathways in the cells with oxidative injury. After pretreating HUVECs with different concentrations of AEE for 24 h in the absence of H_2_O_2_, four potential biomarkers were selected in the cell and cell culture supernatant samples (Table 2), which were involved in valine, leucine and isoleucine biosynthesis; aminoacyl-tRNA biosynthesis; thiamine metabolism; pantothenate and CoA biosynthesis; phenylalanine, tyrosine and tryptophan biosynthesis; pyruvate metabolism; propanoate metabolism; ubiquinone and other terpenoid-quinone biosynthesis; nitrogen metabolism; valine, leucine and isoleucine degradation; phenylalanine metabolism; cysteine and methione metabolism; and tyrosine metabolism (Figure 6A). In the H_2_O_2_-induced cell and cell culture supernatant samples, seven potential biomarkers were selected according to the aforementioned threshold (Table 3) and were involved in cysteine and methionine metabolism, riboflavin metabolism, biotin metabolism, lysine degradation, lysine biosynthesis, N-glycan biosynthesis, glutathione metabolism, aminoacyl-tRNA biosynthesis and tryptophan metabolism (Figure 6B). These results showed that there were few differential metabolites in different treatment groups, but they were involved in many metabolic pathways, which were attributed to the fact that one metabolite might hit many metabolic pathways and only one metabolite in many metabolic pathways. Therefore, many relevant metabolic pathways were only speculative, while many of these differential metabolites, such as, glutathione, riboflavin and l-valine, were relevant to endothelial repair, proliferation and anti-oxidation.

## 3. Discussion

The vascular endothelium, a biological semipermeable membrane between the blood and underlying tissues, has been regarded as an active endocrine, paracrine, and autocrine organ [1,2]. It affects vascular hemostasis by regulating platelet function, the coagulation system, and fibrinolysis [26,27,28,29]. Many cardiovascular diseases are closely related to the dysfunction of the vascular endothelium [30,31,32]. In the present study, the H_2_O_2_-induced oxidative stress model and HFD-induced atherosclerosis model were used to explore the effect of AEE on vascular endothelial cell dysfunction. HFD-fed rats and H_2_O_2_-induced vascular endothelial cells are rapid, invaluable and sensitive models of evoked vascular endothelial dysregulation in vivo and in vitro, respectively [33,34,35,36]. In vitro, after exposure to 200 µM H_2_O_2_ for 22 h, the percentage of early apoptotic HUVECs and E-selectin expression in HUVECs were significantly increased. Notably, the changes in the H_2_O_2_-induced oxidative stress model were significantly ameliorated by the AEE treatment.

AEE is synthesized by combining aspirin with eugenol based on the prodrug principal [11]. AEE exhibits significantly reduced side effects and stronger pharmacological anti-thrombotic, anti-atherosclerotic, and anti-oxidant activities than aspirin and eugenol [12,13,37]. In the present study, AEE significantly decreased the expression of VCAM-1 and E-selectin, and decreased the adherence of THP-1 cells to H_2_O_2_-induced HUVECs. Vascular cell adhesion molecule, a biomarker of endothelial dysfunction, appears to be an important mediator of the development of atherosclerosis. The overexpression of adhesion molecules in the endothelium mediates the adhesion of monocytes, lymphocytes, eosinophils, and basophils to the surface of the vascular endothelium, causing atherosclerosis [38,39,40]. AEE decreased VCAM-1 and E-selectin expression, which might be responsible for the anti-atherosclerotic effects. Cellular metabolomics was performed in the present study to further explore the mechanism by which AEE reduced the expression of VCAM-1 and E-selectin. Many putative mechanisms for endothelial adhesion molecule dysfunction have been well reviewed [41]. Multiple metabolic pathways were involved in vascular endothelial dysfunction. These findings will contribute to further improving our understanding of the underlying mechanism regulating adhesion molecule levels by exploring the effects of AEE on the metabolic pathways in vascular endothelial cells.

Cellular metabolomics is an invaluable and sensitive approach for detecting alterations in metabolic pathways in response to various stimuli by investigating the levels of endogenous small molecule metabolites [19]. In the present study, the results of cell and cell culture supernatant metabolomics analyses identified four potential biomarkers in the cell and cell culture supernatant samples from HUVECs pretreated with AEE in the absence of H_2_O_2_ compared with the control group. These biomarkers are involved in amino acid metabolism, carbohydrate metabolism, energy metabolism, and metabolism of cofactors and vitamins. The levels of 5’-methylthioadenosine, an intermediate metabolite of l-methionine, were significantly increased in the AEE treatment groups. 5’-Methylthioadenosine is a potent A1 receptor agonist that participates in inhibiting the apoptosis of vascular endothelial cells by activating the A1 receptor [42]. After pretreating HUVECs with AEE in the absence of H_2_O_2_, the levels of l-tyrosine and l-valine were also markedly increased. l-Valine exerts many beneficial effects, including repairing tissues and increasing energy [43,44]. Researchers have speculated that AEE might enhance the endothelial repair capacity by modulating methionine and valine metabolism. After treating HUVECs with H_2_O_2_, the results of biomarker and pathway analyses identified eight biomarkers in the cell and cell culture supernatant samples. These biomarkers are involved in riboflavin metabolism, *N*-glycan biosynthesis, and glutathione metabolism. Several of these biomarkers play pivotal roles in the pathways regulating vascular homeostasis. *N*-Acetylaspartylglutamic acid, glutathione, l-lysine, and riboflavin effectively protect the vascular endothelium by inhibiting the adhesion of leukocytes to endothelial cells, maintaining the blood lipid balance, reducing oxidative stress, and enhancing anti-oxidant and vascular repair capabilities [45,46,47,48,49,50]. Indole acetaldehyde, a tryptophan metabolite, inhibits endothelial cell proliferation [51]. Notably, after the AEE treatment, the changes in the H_2_O_2_-induced HUVECs showed a reversible trend to a physiological level. These results of cellular metabolomics as analyses and our previous findings from plasma and urine metabonomics in HFD-fed hamsters [17] suggested that the mechanism underlying the anti-atherosclerotic effects of AEE might be related to a decrease in vascular endothelial dysfunction by ameliorating metabolic changes, increasing the anti-oxidative stress activity and decreasing adhesion molecule expression. The HFD-induced atherosclerosis model was utilized in vivo to further confirm the cytoprotective effects of AEE.

After treating rats with HFD for 12 weeks, VCAM-1 and E-selectin expression in the aorta and the levels of sVCAM-1 and sE-selectin were increased significantly. Additionally, the levels of LDL, HDL, TCH, and AGE significantly deviated from physiological levels, and the trends in LDL, HDL, and TCH levels were consistent with previous studies [15,37]. These findings confirmed that endothelium in HFD-fed rats had been disturbed. Excessive LDL, particularly oxidized low-density lipoproteins (ox-LDL), causes early atherosclerosis by disturbing the function of the endothelium [52,53,54]. Moreover, the formation of AGEs induced by various stimuli accelerates the development of atherosclerosis by increasing the expression of adhesion molecules [55,56,57]. In the present study, AEE reduced the increase in the serum LDL, AGE, sVCAM-1 and sE-selectin levels in HFD-fed rats and decreased VCAM-1 and E-selectin expression, which might be related to an amelioration of the excessive accumulation of LDL and AGEs in the blood vessel. Based on the results from the in-vivo and in-vitro experiments, the anti-atherosclerotic mechanism of AEE might be related to a decrease in vascular endothelial dysfunction through the amelioration of metabolic changes, an increase in the anti-oxidative stress activity, and a decrease in adhesion molecule expression. Our previous study had also confirmed that AEE could significantly enhance the anti-oxidative stress ability of vascular endothelial. It was well known that oxidative stress could stimulus the adhesion molecule expression on the vascular endothelial cells. Therefore, we proposed that the inhibiting effect of AEE on adhesion molecules was related to reduce the oxidative stress of vascular endothelium

Due to the limitations of the UPLC/QTOF-MS method for metabolomics analyses, many differentially altered metabolites are unable to be identified and thus some important biological information is missed. In the present study, the findings of the metabolomics analysis based on UPLC/QTOF-MS only partially explained the biological changes among the different treatment groups. Certainly, further studies of how AEE mutually affects these metabolic pathways are needed.

## 4. Materials and Methods

### 4.1. Chemicals

Transparent AEE crystals with a purity of 99.5% according to RE-HPLC were prepared in the Lanzhou Institute of Husbandry and Pharmaceutical Sciences of CAAS. The H_2_O_2_ solution (cat number: 323381), dimethyl sulfoxide (DMSO) and Trypsin-EDTA were supplied by Sigma (St. Louis, MO, USA). Deionized water (18.25 MΩ) was prepared with a Direct-Q^®^3 system (Millipore, Bedford, MA, USA). The Annexin V/PE apoptosis detection kit was purchased from BD Biosciences (San Jose, CA, USA). Anti-ICAM-1, anti-VCAM-1, and anti-E-selectin antibodies were obtained from Abcam (Cambridge, MA, USA). The rat-soluble vascular cell adhesion molecule 1(sVCAM-1) ELISA kit, rat soluble intercellular adhesion molecule 1 (sICAM-1) ELISA kit, and rat soluble E-selectin (sE-selectin) ELISA kit were obtained from Elabscience (Wuhan, China); the advanced glycation end product (AGE) ELISA kit was purchased from Wuhan USCN Business Co., Ltd. (Wuhan, China). MS-grade acetonitrile was purchased from Thermo Fisher Scientific (Waltham, MA, USA). Carboxymethylcellulose sodium (CMC-Na) was supplied by Tianjin Chemical Reagent Company (Tianjin, China)

### 4.2. Cell Culture and Treatments

Human umbilical vein endothelial cells (ATCC^®^ CRL-4053™) purchased from ATCC (Rockville, MD, USA) were cultured in cell culture flasks with DMEM/F12 (1:1) supplemented with 10% fetal bovine serum. The media were refreshed once every two days. Subcultures were performed with trypsin-EDTA. Experiments were subsequently conducted on cells at passages 6–7.

HUVECs were randomly divided into three groups: a normal group, model group and AEE pretreatment group. Cells in the normal group were incubated with the culture medium. The model group was incubated with the culture medium containing 200 µM H_2_O_2_ for 22 h. In the AEE pretreatment groups, cells were pre-incubated with culture medium containing different concentrations of AEE (0.5, 1, or 2 µM) for 24 h and then incubated with medium containing 200 µM H_2_O_2_ for 22 h.

### 4.3. Apoptosis Detection Using Flow Cytometry

The apoptosis of HUVECs in the different treatment groups was quantified with an Annexin V/PE apoptosis detection kit using flow cytometry. Briefly, the cells were collected and washed three times with cold PBS. Next, cells were incubated with PE-Annexin V and stained with 7-ADD for 20 min at room temperature in the dark. The double-stained cells were analyzed using flow cytometry, and the following controls were used to establish compensation and quadrants: unstained cells, cells stained with PE-Annexin V and cells stained with 7-ADD.

### 4.4. Adhesion Assay

HUVECs were cultured in 96-well flat-bottom plates (0.1 mL/well) at a density of 1 × 105 cells/mL for 24 h. Cells were then pretreated with the different concentrations of AEE (0.5, 1, or 2 µM) for 24 h, followed by 200 µM H_2_O_2_ for 22 h. The culture medium was then removed, and the THP-1 cells prelabeled with BECF-AM were cocultured with the treated HUVECs at 37 °C for 1 h in a 5% CO_2_ incubator. The non-adherent cells were removed by gentle aspiration. Plates were washed three times with DMEM/F12 (1:1). The number of adherent cells was estimated under a microscope, and then cells were lysed with 0.1 mL of 0.25% Triton X-100. The fluorescence intensity was measured at an excitation wavelength of 485 nm and an emission wavelength of 538 nm using an Enspire Microplate Reader (PerkinElmer, Waltham, MA, USA).

### 4.5. Adhesion Molecule Expression

HUVECs were grown to confluence, pretreated with AEE for 24 h and stimulated with 200 µM H_2_O_2_ for 22 h to determine whether AEE modified the H_2_O_2_-induced expression of adhesion molecules. At the end of the stimulation period, HUVECs were harvested, incubated with anti-VCAM-1, anti-ICAM-1, and anti-E-selectin antibodies for 1 h at room temperature, and then incubated with an Alexa Fluor 488-labeled secondary antibody. After HUVECs were washed three times, their immunofluorescence intensity was analyzed using flow cytometry.

### 4.6. Cellular Metabolite Extraction

After incubation, the cell culture supernatant was collected, centrifuged at 800 rpm for 10 min at 4 °C and the supernatant was transferred into clean tubes. Twenty microliters of supernatant from each sample were mixed to prepare a quality control (QC) sample. Then, the mixture was divided into aliquots with the same volume as other samples and prepared using the method described below. Methanol was added to the supernatant (3:1, *v*/*v*), vortexed for 1 min, incubated at 4 °C for 30 min, and centrifuged at 14000 g for 10 min at 4 °C. The supernatant was transferred to a new tube and dried with a stream of nitrogen. The residue was resuspended in 500 µL of methanol/water (3:1, *v*/*v*) and then filtered through a 0.2-µm nylon mesh into sample vials.

After collecting the supernatant from cultured cells, the cells were quickly quenched by adding 80% methanol (*v*/*v*, cooled to −80 °C), incubating the mixture at −80 °C for 5 min, and scraping the cells from the cell culture plate. Then, the mixture was lysed with two freeze-thaw cycles (frozen in liquid nitrogen and thawing at 37 °C), mixed for 1 min, and pelleted by centrifugation at 14000× *g* for 10 min at 4 °C. Twenty microliters of supernatant from each sample were mixed to prepare a quality control (QC) sample. Then, the mixture was divided into aliquots with the same volume as other samples and prepared together. The supernatant was filtered through a 0.2-µm nylon mesh into sample vials.

### 4.7. UPLC/QTOF-MS Analysis of Cellular Metabolites

The metabolomics analysis was performed with an Agilent 1290 Infinity LC system coupled to an Agilent 6530 Accurate-mass Q-TOF mass spectrometer (Agilent Technologies, Palo Alto, CA, USA). Chromatographic separation of cell and cell culture supernatant samples was performed on an Agilent ZORBAX SB-C18 threaded column (2.1 × 150 mm, 1.8 μm, Agilent Technologies, Palo Alto, CA, USA) maintained at 35 °C. The mobile phase consisted of solvent A (0.1% formic acid in water, *v*/*v*) and B (0.1% formic acid in acetonitrile, *v*/*v*). The optimized gradient program was established. The post time was set to 3 min for equilibration. Mass spectrometry was performed in both positive (ESI+) and negative (ESI−) electrospray ionization modes. The fragment voltage was set to 135 V and the skimmer voltage was set to 65 V. The capillary voltages were set to 4.0 KV in positive mode and 3.5 KV in negative mode. The drying gas (nitrogen) was used at a flow rate of 10 L/min at 350 °C and the nebulizer pressure was set to 45 psig. Data were collected in centroid mode from 50–1000 m/z using an extended dynamic model.

The raw MS data were initially processed with the Mass Profiler Professional (MPP) software (Agilent Technologies, USA) to filter noise, correct the baseline, align peaks, and identity and quantify peaks. The match tolerance of mass span is 10 ppm, and the match tolerance of retention time’s span is 0.10 min. The obtained data were imported into SIMCA-P (version 13.0, Umetrics AB, Umea, Sweden), where a principal component analysis (PCA) and partial least squares discriminant analysis (OPLS-DA) were performed on the dataset. The quality of OPLS-DA models was described by R2X, R2Y, and Q2, and its validity was evaluated by performing permutation testing (with 200 permutations). The variable importance in the projection (VIP > 1) value of the validated OPLS-DA model and the *p* values from one-way ANOVA (*p* < 0.05) were used as the measurement indices to select potential metabolites. Metabolites were identified through a mass-based search followed by manual verification. Accurate mass values of the molecular ions of interest in TOF-MS data were searched against METLIN and Human Metabolome Database (HMDB). Then, an MS/MS analysis was conducted to confirm the structure of potential biomarkers by matching the masses of the fragments. The parent ion mass tolerance is ±10 ppm and mass/charge (m/z) of products tolerance is ±10 ppm. The clustering analysis of the potential biomarkers and pathway analysis were performed using MetaboAnalyst 3.0 [58] and the metabolic pathways were identified using the KEGG database.

### 4.8. Animal Experiment

Thirty male Sprague-Dawley (SD) rats (6 weeks old) weighing 120–130 g were purchased from the Laboratory Animal Center of Lanzhou Veterinary Research Institute (Lanzhou, China). All animals were housed in groups in the facilities at a controlled relative humidity (45–65%) and temperature (22 ± 2 °C). Feed and drinking water were supplied to the SD rats ad libitum. The rats were randomly assigned into three groups (*n* = 10): (1) the control group, in which rats were fed the normal diet; (2) the high-fat diet (HFD) group, in which rats were fed the HFD; and (3) the AEE group, in which rats were simultaneously fed the HFD and AEE (54 mg/kg body weight). The normal diet (12.3% lipids, 63.3% carbohydrates and 24.4% proteins) was purchased from Keao Xieli Feed Co., Ltd. (Beijing, China) and the HFD (41.5% lipids, 40.2% carbohydrates and 18.3% proteins) was customized from Keao Xieli Feed Co., Ltd. The dose of AEE was selected according to previous studies from our group [37,59]. Rats were sacrificed by injecting pentobarbital (30 mg/kg body weight). Blood samples were collected from the heart into heparin-treated vacuum tubes. Plasma samples were obtained after the centrifugation of blood at 3500 rpm for 10 min at 4 °C and stored at −80 °C until analysis. The aortas were carefully isolated from rats and fixed with 4% formalin for pathological observations. All experimental protocols and procedures were approved by the Institutional Animal Care and Use Committee of Lanzhou Institute of Husbandry and Pharmaceutical Science of Chinese Academy of Agricultural Sciences (Approval No. NKMYD201805; Approval Date: 18 October 2018). Animal welfare and experimental procedures were performed strictly in accordance with the Guidelines for the Care and Use of Laboratory Animals issued by the US National Institutes of Health.

### 4.9. Measurement of Serum AGE Levels

Serum AGE levels were detected using an enzyme-linked immunosorbent assay kit for AGEs according to the manufacturer’s protocols.

### 4.10. Adhesion Molecule Expression in the Aorta

Aortas were formalin-fixed, quick-frozen in liquid nitrogen, sectioned, and antigens were retrieved to investigate the expression of VCAM-1, ICAM-1, and E-selectin in the aorta. Sections were incubated with primary antibodies against VCAM-1, ICAM-1, and E-selectin. Then, sections were incubated with an Alexa Fluor 488-labeled goat anti-rabbit antibody using the standard protocol. Moreover, the aortas that were only incubated with the Alexa Fluor 488-labeled goat anti-rabbit antibody served as the negative control. The result was observed and analyzed with a laser scanning confocal microscope and Zen blue version 2.3 software (ZEISS LSM-800, Jena, Germany).

### 4.11. Serum Levels of Adhesion Molecules

Serum levels of sVCAM-1, sICAM-1 and sE-selectin were detected with the indicated ELISA kits according to the manufacturer’s protocols.

### 4.12. Statistical Analysis

Statistical analyses were performed using SAS 9.2 software (SAS Institute Inc., Cary, NC, USA). All data are presented as the means ± SD. The differences among groups were analyzed with one-way analysis of variance (ANOVA). When significance was achieved, Duncan’s multiple comparisons test was conducted to identify the sources of differences. Statistical significance was considered at *p* < 0.05.

## 5. Conclusions

AEE effectively protected the vascular endothelium by enhancing anti-oxidant and vascular repair capabilities and inhibiting the adhesion of leukocytes to endothelial cells. The inhibiting effect of AEE on adhesion molecules was related to reduced oxidative stress of vascular endothelial by ameliorating the vascular endothelium metabolism.

## Figures and Tables

**Table 1:** The effects of AEE on serum biochemical indices and AGE levels.

Variables	Normal Group	HFD Group	AEE Group
HDL (mmol/L)	0.96 ± 0.10	0.67 ± 0.06 *	0.79 ± 0.02 ^#^
LDL (mmol/L)	0.53 ± 0.06	0.80 ± 0.19 *	0.68 ± 0.04 ^#^
TCH (mmol/L)	1.24 ± 0.10	2.41 ± 0.48 *	1.58 ± 0.34 ^#^
AGE (ng/mL)	390.38 ± 18.13	786.64 ± 49.20 *	497.04 ± 34.70 ^#^

HDL: high density lipoprotein; LDL: low density lipoprotein; TCH: total cholesterol; HFD: high-fat diet; AGE: advanced glycosylation end product. Values are presented as the means ± SD where applicable (*n* = 8). * *p* < 0.05 compared with the normal group; ^#^ *p*< 0.05 compared with the HFD group.

**Table 2:** The result of biomarkers identified in the cells and cell supernatants treated with AEE in the absence of H_2_O_2._

No.	RT	VIP	Formula	Metabolite	SM	m/z	Fold Change
							AEE/C
1	3.14	3.39	C_9_H_11_NO_3_	l-Tyrosine	−	181.07285	2.16 *
2	1.49	1.01	C_5_H_11_NO_2_	l-valine	+	117.07925	3.17 *
3	10.56	2.19	C_7_H_12_O_5_	2-Isopropylmalic acid	+	176.06818	0.49 *
4	5.7	4.093	C_11_H_15_N_5_O_3_S	5’-Methylthioadenosine	+	297.08875	1.75 *

RT: retention time; VIP: variable importance in the projection; SM: scan mode; +: metabolites identified in positive mode; −: metabolites identified in negative mode. * *p* < 0.05 compared with the normal group; AEE/C: AEE group compared with the normal group.

**Table 3:** The effect of AEE on the levels of potential biomarkers in cell and cell supernatants stimulated with H_2_O_2._

No.	RT	VIP	Formula	Metabolite	SM	m/z	Fold Change	
							C/H_2_O_2_	AEE/H_2_O_2_
1	1.46	1.45	C_10_H_17_N_3_O_6_S	Glutathione	+ −	307.08353	2.78 *	1.40 *
2	1.14	1.27	C_6_H_14_N_2_O_2_	l-Lysine	+	146.10437	6.17 *	5.16 *
3	2.04	4.13	C_11_H_16_N_2_O_8_	*N*-Acetylaspartylglutamic acid	+	304.08963	4.66 *	2.85 *
4	5.7	1.96	C_11_H_15_N_5_O_3_S	5’-Methylthioadenosine	+	297.08932	1.974 *	3.32 *
5	16.9	1.94	C_21_H_39_O_9_P	Dolichyl b-d-glucosyl phosphate	+	466.2366	2.264 *	1.028
6	8.37	2.04	C_17_H_20_N_4_O_6_	Riboflavin	+	376.13806	5.77 *	1.91 *
7	10.51	3.59	C_11_H_20_O_6_	Prenyl glucoside	+	248.12555	2.69 *	1.04
8	5.21	2.29	C_10_H_9_NO	Indole acetaldehyde	+	159.06776	0.17 *	0.69 *

RT: retention time; VIP: variable importance in the projection; SM: scan mode; +: metabolites identified in positive mode; −: metabolites identified in negative mode. Metabolites identified in both positive and negative modes; * *p* < 0.05 compared with the H_2_O_2_ group; C/H_2_O_2_: normal group compared with the H_2_O_2_ group; AEE/H_2_O_2_: AEE group compared with the H_2_O_2_ group.

## Supplementary Materials

Supplementary materials can be found at https://www.mdpi.com/1422-0067/20/13/3165/s1.

## Abbreviations

HUVECs	Human umbilical vein endothelial cells
HFD	High fat diet
VCAM-1	Vascular cell adhesion molecule 1
ICAM-1	Soluble intercellular adhesion molecule 1
MDA	Malondialdehyde
HDL	High density lipoprotein
AGEs	Advanced glycosylation end products
LDL	Low density lipoprotein
TCH	Total cholesterol
PCA	Principal component analysis
OPLS-DA	Orthogonal projections to latent structures discriminate analysis
VIP	Variable importance in the projection
UPLC/QTOF-MS	Ultra-performance liquid chromatography/quadrupole time of flight-mass spectrometry
