# Supplementary material for: Cellular Metabolomics Reveal the Mechanism Underlying the Anti-Atherosclerotic Effects of Aspirin Eugenol Ester on Vascular Endothelial Dysfunction
Source: Int J Mol Sci. 2019 Jun 28;20(13):3165. doi: 10.3390/ijms20133165 (PMC6651823; doi:10.3390/ijms20133165)
Supplement: Supplementary file 1 [file ijms-20-03165-s001.pdf]

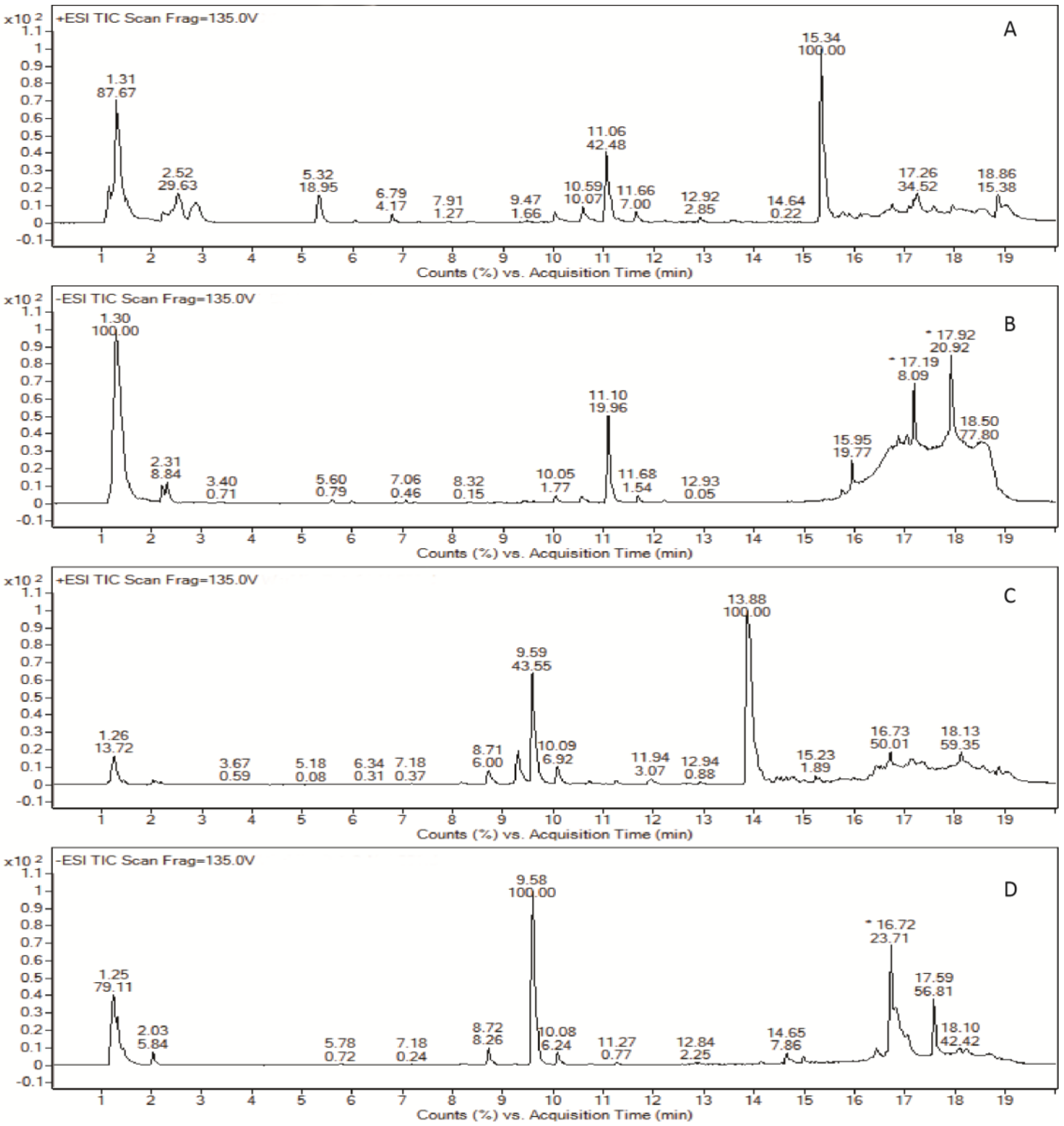

Figure S Representative TICs of cell culture supernatant and cell samples in positive model and negative model. A-B: Representative TICs of cell culture supernatant samples in positive model and negative model. C-D: Representative TICs of cell samples in positive model and negative model.
